# Analysis of the outage performance of energy-harvesting cooperative-NOMA system with relay selection methods

**DOI:** 10.1038/s41598-024-61213-0

**Published:** 2024-05-10

**Authors:** Yulin Zhou, Yang Zhang, Aziz Altaf Khuwaja, Zhao Wang, Qifei Zhang

**Affiliations:** 1 Ningbo Innovation Center, Zhejiang University, Ningbo, 315000 Zhejiang China; 2 School of Software Technology, Zhejiang University, Ningbo, 315000 Zhejiang China; 3 Department of Electrical Engineering, Sukkur IBA University, Sukkur, Sindh 65200 Pakistan

**Keywords:** Engineering, Electrical and electronic engineering

## Abstract

Recent years have witnessed the remarkable progress in wireless communication systems due to the escalating demand for higher data rates, improved reliability, and increased energy efficiency. In this regard, Non-Orthogonal Multiple Access (NOMA) has emerged as a promising technology, enhancing spectral efficiency and accommodating multiple users concurrently within the same time and frequency resources. Simultaneously, the energy harvesting has surfaced as a sustainable solution, converting ambient environmental energy into usable electrical power for operating communication nodes. This paper proposes a cooperative NOMA transmission scheme integrating energy harvesting and utilizing Least Squares (LS) channel estimation for precise Channel State Information (CSI) acquisition. The objective is to establish an optimal communication path from source to destination. Relay selection methods: Optimal Relay Selection (ORS) and Max-Min Relay Selection (MMRS), are compared, focusing on their impact on the system performance. The analysis considers the influence of the number of relays and power allocation factor on the system, with a specific emphasis on the outage probability expressions. Comparative analysis between the cooperative-NOMA and the traditional cooperative relaying without NOMA reveals the superior performance of the cooperative-NOMA. Additionally, the ORS scheme outperforms MMRS in terms of the outage performance.

## Introduction

Non-orthogonal multiple access (NOMA) has gained significant attention in the field of wireless communication^1^. NOMA departs from traditional orthogonal multiple access schemes by allowing multiple users to share the same time-frequency resources. This approach relies on power domain multiplexing, allocating different power levels to different users. The result is a more efficient use of available spectrum^2^, enabling higher throughput and improved connectivity in wireless networks. Notably, NOMA exhibits compatibility with the cooperative communications^3–5^. In the context of cooperative NOMA, various aspects of this technology have been explored such as, NOMA users with the best Channel State Information (CSI) engage in cooperation^6^. Furthermore, scenarios involving multiple relays with finite energy storage capability, specifically within the massive Internet of Things (IoT) systems have been investigated^7^. Additionally, a novel transmission method in hybrid visible light communications (VLC)/radio-frequency (RF) systems has been proposed^8^. However, despite these advancements, it is crucial to note that the aforementioned systems do not address the issue of relay selection.

In traditional communication networks, the relay selection methods have been extensively studied due to their inherent attributes of the superior performance and realizing the performance gain of multi-antenna and multi-hop transmissions^9–11^. There are various relay selection methods in NOMA, and the choice of method depends on the specific system requirements and optimization criteria. Notably, the Optimal Relay Selection (ORS) method was introduced in the context of amplify-and-forward (AF) cooperative communication with full-duplex (FD) operation^11^. In another scenario, the Max-Min Relay Selection (MMRS) method finds application in AF cooperative diversity systems^12^. Factors such as fairness, energy efficiency, and system throughput will influence the selection of an appropriate relay selection algorithm in NOMA systems.

In previous studies, a comprehensive analysis of multi-user relay cooperative transmission within NOMA systems has been lacking. The distinctive characteristics of carrier signals and channel compositions in NOMA systems render existing estimation methods unsuitable for application in cooperative NOMA systems. In the uplink power-domain NOMA, clustering techniques have been applied for channel estimation without requiring the pilot symbols^13^. Additionally, a novel semi-blind channel estimation method has been proposed^14^. Also, a user activity detection and channel estimation of neural network for grant-free NOMA has been introduced^15^. It is worth noting that these prior works, while valuable, were not conducted within the framework of cooperative NOMA systems.

Motivated by the above challenges, we aim to investigate an energy-harvesting cooperative-NOMA-based relaying network with relay selection. The primary contribution of this study lies in the integration of NOMA, cooperative relaying, and relay selection within the proposed scheme. We specifically focus on scenarios where each relay operates in one of two power states: either at full capacity (maximum power) or not active (zero power), enabling the selection of the optimal relay node. To enhance the accuracy of CSI, we employ the Least Squares (LS) channel estimation scheme. Moreover, we employ two relay selection methods, ORS, and MMRS, within the cooperative-NOMA system. To compare their performance, we utilize outage probability metrics. The numerical result shows that the cooperative NOMA performs better than the cooperative relaying without NOMA. Furthermore, the ORS method outperforms the MMRS scheme.

## Results

In this section, we compare the performance of two relay selection methods under the proposed cooperative-NOMA system model and analyze the outage probability under different parameter changes. For all simulations, we assume that the distance between the source and destination is a fixed value, and the relay positions are randomly distributed around the source following a Poisson distribution. The abscissa represents the transmission power, and the ordinate represents the outage probability. The parameter settings are as follows: $R_0=2, \alpha _{SR}=0.1, \beta _{SR}=0.1, e=2$, and $a_1=0.5$. All channels are generated as i.i.d. complex Gaussian random variables with zero-mean and unit-variance. The noises at the relays and receiver are also i.i.d. complex Gaussian random variables with zero-mean and unit-variance.

**Figure 1:**
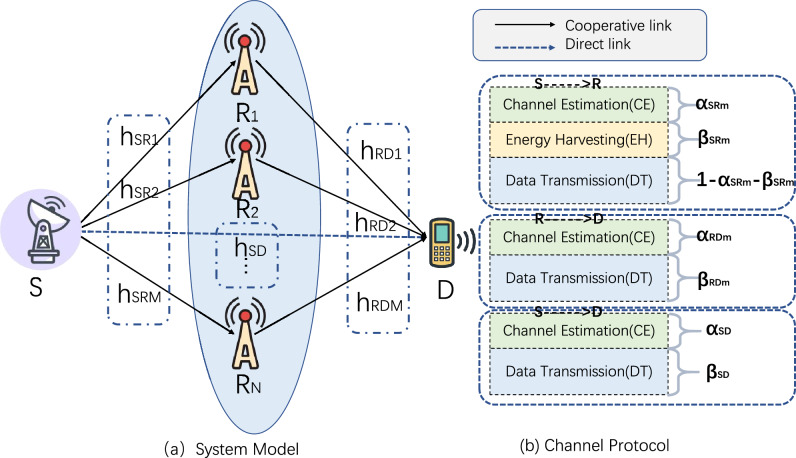
System model.

**Figure 2:**
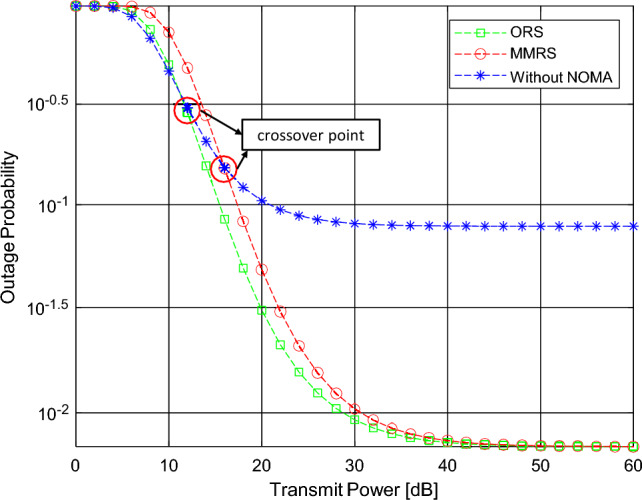
Outage probability versus the transmitted power $P_s$.

Figure 2 illustrates the outage probability as a function of $P_s$ for different relay selection schemes in a simulation. As depicted in the figure, the outage probability of ORS and MMRS is lower than the case without NOMA at $P_s = 12$dB and $P_s = 17$dB, respectively. The first significant observation is that all the relay selection policies converge to an error floor, providing zero diversity gain. Additionally, the two relay selection schemes based on NOMA outperform those without NOMA, confirming the enhanced channel performance due to NOMA. As anticipated, ORS performs better than MMRS. This difference arises because ORS takes into account the coupling of two stages of SNR, whereas MMRS only considers a single channel and does not integrate the performance of the entire channel.

**Figure 3:**
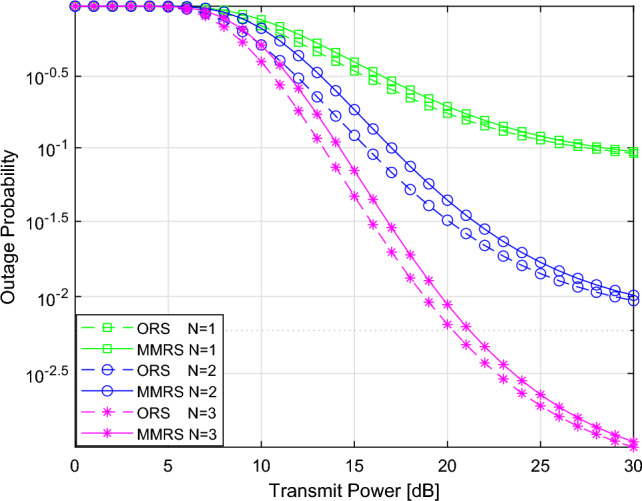
Outage probability versus the transmitted power with the change of *N*.

Figure 3 illustrates the influence of the number of relays on the outage probability. Firstly, for the ORS and MMRS schemes with a larger N, the system’s outage performance is better because increasing the number of relays in a cooperative-NOMA network provides diversity and spatial multiplexing, which contribute to enhanced system performance and reliability. In addition, it can be found that with the increment in *N*, the performance improvement of ORS is greater than that of MMRS.

**Figure 4:**
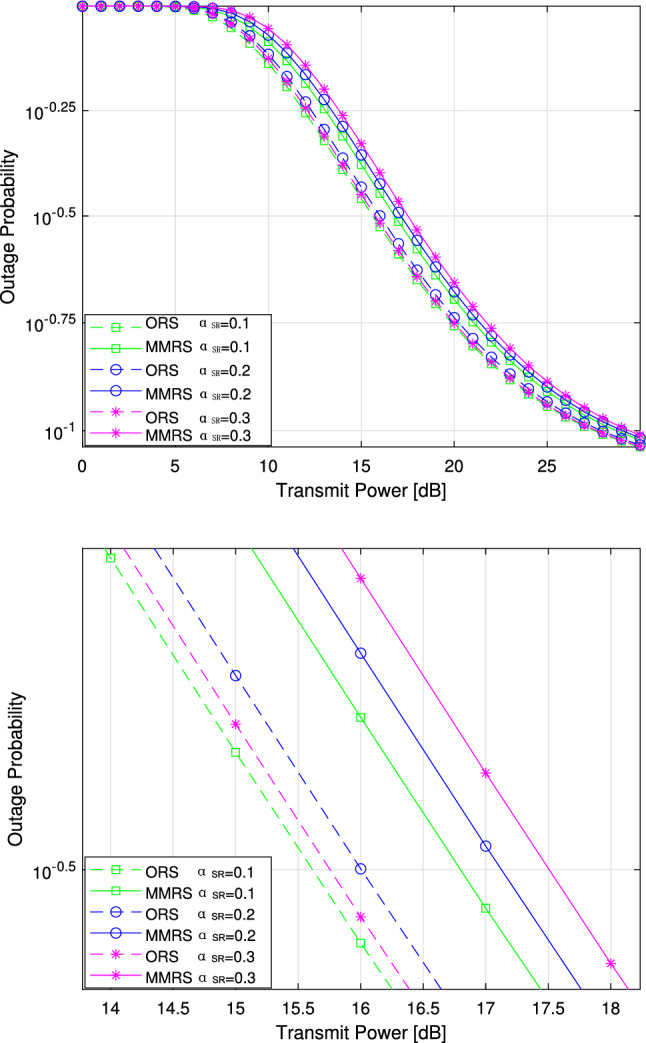
Outage probability versus the transmitted power with the change of $\alpha _{SR}$.

We also demonstrate the performance variation of the parameter $\alpha _{SR}$ in Fig. 4. As shown in Fig. 4a, the outage probability initially remains stable with an increasing transmit power, followed by a rapid increase. In Fig.4(b), which is a localized version of Fig. 4a, we observe a negative relationship between the increase in $\alpha _{SR}$ and the outage probability of the cooperative system. In our parameter design, the improvement of $\alpha _{SR}$ does not impact $\beta _{SR}$, but it affects the proportion of data transmission in the transmission protocol. This could be a possible cause for the increase in outage probability.

**Figure 5:**
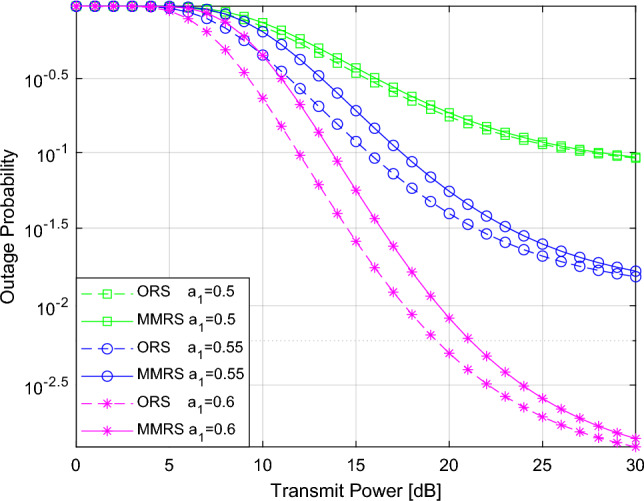
Outage probability versus the transmitted power with the change of $a_1$.

Finally, Fig. 5 illustrates the outage probability as a function of the transmit power $P_s$ for various power allocation coefficients $a_1$. The variation in $a_1$ notably impacts the reduction of the outage probability. Furthermore, the relay selection method consistently reduces the outage probability, irrespective of $a_1$ changes. The ORS scheme consistently outperforms the MMRS scheme in all scenarios.

## Discussion

In this paper, we investigated an innovative energy-harvesting cooperative-NOMA network with channel estimation, where communication is facilitated through a selected relay among randomly distributed relays. The incorporation of NOMA enhances remote destination reliability without consuming extra energy, achieved through energy-harvesting assistance, which reduces outage probability and mitigates high outage risks at the remote destination. Our approach addresses channel estimation and relay selection in the relay network under the AF protocol, ensuring accurate CSI in the cooperative-NOMA system. We compared two relay selection criteria: ORS and MMRS, finding that both methods significantly reduce system outage probability, with ORS slightly outperforming MMRS. Simulation results indicated that NOMA integration enhances spectral efficiency and access volume. Furthermore, the ORS method, incorporating both forward and backward channels, proved superior to MMRS.

## Methods

Figure 1 depicts a NOMA-based cooperative network consists of a single source (S), single destination (D), and *N* number of relays ($\text {R}_{N}$). We assume that the source, destination and relays are only equipped with one antenna which can be used for both transmission and reception to simplify the analysis and provide clear, fundamental insights into the behavior of the system under study. In the proposed network, relays harvest energy from the source and forward the information to the destination using the harvested energy as their transmit powers. The AF strategy is applied at relays and we consider both the direct transmission link from source to destination and the cooperative transmission. For each device, we use $h_{SR}$, $h_{RD}$, $h_{SD}$ to represent the channel coefficients from source to relays, relays to destination, and source to destination, respectively.

### Cooperative-NOMA transmission

During the first phase, the source broadcasts a composite signal as

1 $$\begin{aligned} x(t_1)=\sqrt{a_1 P_s} x_1(t_1) +\sqrt{a_2 P_s} x_2(t_1). \end{aligned}$$

where $x_1(t_1)$ and $x_2(t_1)$ are the signals for relay and destination, respectively, by appropriately designing the power levels of pilot and data signals, it can be ensured that channel estimation can be performed while effective energy conversion is being carried out. and with $E[x_1(t_1)]=E[x_2(t_1)]=1$, $P_s$ is the transmit power, $x_1[i]$ is the transmitted signal to relay, $a_1$ and $a_2$ the power coefficients for signal send to the relay node and the destination, respectively.

Moreover, the transmission protocol is also shown in Fig. 1, the channel between the source and the relay includes three parts: Channel Estimation (CE), Energy Harvesting (EH) and Data Transmission (DT).

The observations at the relays of the signal used for CE, EH and DT are given as

2a $$\begin{aligned} y_{SR_m}\left[ i\right]&=\frac{h_{SR_m}}{\sqrt{1+d_{SR_m}^e}}{\sum _{k=1}^{2}{\sqrt{P_sa_k\alpha _{SR}}x_k\left[ i\right] }}+n_1\left[ i\right] , \end{aligned}$$

2b $$\begin{aligned} y_{SR_m}\left[ j\right]&=\frac{h_{SR_m}}{\sqrt{1+d_{SR_m}^e}}{\sum _{k=1}^{2}{\sqrt{P_sa_k\beta _{SR}}x_k\left[ j\right] }}+n_1\left[ j\right] , \end{aligned}$$

2c $$\begin{aligned} y_{SR_m}\left[ p\right]&=\frac{h_{SR_m}}{\sqrt{1+d_{SR_m}^e}}{\sum _{k=1}^{2}{\sqrt{P_sa_k\gamma _{SR}}x_k\left[ p\right] }}+n_1\left[ p\right] , \end{aligned}$$

where $h_{SR_m}$ represents the channel coefficients from source to $m^{\text {th}}$ relay, $\alpha _{SR}$ is the proportion of CE in a single time slot, $\beta _{SR}$ is the proportion of CE, $1-\alpha _{SR}-\beta _{SR}$ is the proportion of DT, $d_{SR_m}$ is the distance from source to $m^{\text {th}}$ relay, *e* is the channel large-scale fading coefficient, $n_1[i]\sim \mathscr{C}\mathscr{N}\left( 0,\sigma ^2\right)$ is the Gaussian noise. The symbol (CN) stands for “Complex Normal” .

The direct link signal received by the destination from the source can be expressed as

3 $$\begin{aligned} {y_{SD}=\frac{h_{SD}}{\sqrt{1+d_{SD_m}^e}}\sum _{k=1}^{2}{\sqrt{P_sa_k\beta _{SD}}x_k\left[ i\right] }+n_3.} \end{aligned}$$

where $\beta _{SD}$ is the proportion of DT in a single time slot from source to destination.

To simplify the analysis, we assume that the harvested energy scales linearly with the input power with the rectifier efficiency $\eta \in (0, 1]$. Thus, from Eq. (2b) the harvested energy in *T* times can be expressed as

4 $$\begin{aligned} P_R=\frac{\eta P_s\left| h_{SR_m}\right| ^2\beta _{SR} T}{1+d_{SR_m}^e}. \end{aligned}$$

The relay amplifies the received signal and then forwards it to the destination by using the harvested energy, the signal received at the destination from the relay can be expressed as

5 $$\begin{aligned} y_{RD_m}&=\frac{Gh_{RD_m}}{\sqrt{1+d_{RD_m}^e}}\sqrt{P_R\beta _{RD}}y_{SR_m}\left[ p\right] +n_2 . \end{aligned}$$

where $d_{RD_m}$ is the distance from relay to destination, $G=\sqrt{\frac{P_R}{P_s\frac{\left| h_{SR_m}\right| ^2}{1+d_{SR_m}^e}+\sigma _1^2}}$ is the amplifying coefficient of AF transmission protocol.

Based on Eqs. (4) and (5), the received SNR for the destination to detect the signal forwarded from the relay is given by

6 $$\begin{aligned} \gamma _{RD_m}=\frac{\eta ^2P_s^2\beta _{SR}^2\rho \left| h_{RD_m}\right| ^2a_1(1-\alpha _{SR}-\beta _{SR})}{\Gamma _{A}+\Gamma _{B}+\Gamma {C}}. \end{aligned}$$

where $$\Gamma _{A}=\eta ^2P_s^2\beta _{SR}^2\rho \left| h_{RD_m}\right| ^2a_2(1-\alpha _{SR}-\beta _{SR})$$, $$\Gamma _{B}=\eta P_s\beta _{SR}\frac{\left| h_{RD_m}\right| ^2}{\left| h_{SR_m}\right| ^2}{(1+d_{SR_m}^e)}^2$$, and $$\Gamma _{C}=\frac{(P_s+\sigma _1^2)}{\left| h_{SR_m}\right| ^2}{(1+d_{SR_m}^e)}^2(1+d_{RD_m}^e)$$.

We consider the imperfect successive interference cancellation (SIC) conditions. In this case, it is considered that the destination does not have perfect knowledge of the relay signal information. Destination proceeds to decode signal $x_1(t_1)$ using SIC technique. The received SINR at the destination to detect signal $x_1(t_1)$ is given by $\gamma _{x_1}=\gamma _{RD_m}$. After performing SIC successfully, the received SNR at the destination to detect signal $x_2(t_1)$ is given by

7 $$\begin{aligned} \gamma _{x_2}=\frac{\eta ^2P_s^2\left| h_{SR_m}\right| ^4\beta _{SR}^2\rho \left| h_{RD_m}\right| ^2a_1(1-\alpha _{SR}-\beta _{SR})}{\sigma _1^2(1+d_{RD_m}^e)(1+d_{SR_m}^e)}. \end{aligned}$$

### Channel estimation

By using the LS channel estimation method to estimate the CSI, we apply the LS channel estimation method^16^ to ${{\hat{h}}}_{SD}$, ${{\hat{h}}}_{RD}$ and ${{\hat{h}}}_{SR}$ to get

8a $$\begin{aligned} {{\hat{h}}}_{SD}&=h_{SD}+\frac{n_3\sqrt{1+d_{SD}^e}}{\sum _{k=1}^{K}{\sqrt{P_sa_k}x_k\left[ i\right] }}, \end{aligned}$$

8b $$\begin{aligned} {{\hat{h}}}_{RD}&=h_{RD}+\frac{n_2\left[ i\right] \sqrt{1+d_{RD}^e}}{G\sqrt{P_R}y_{SR}\left[ p\right] }, \end{aligned}$$

8c $$\begin{aligned} {{\hat{h}}}_{SR}&=h_{SR}+\frac{n_1\left[ p\right] \sqrt{1+d_{SR}^e}}{\sum _{k=1}^{K}{\sqrt{P_sa_k\alpha }x_k\left[ p\right] }}. \end{aligned}$$

To denote the estimation error following the relation ${{\widetilde{e}}}_{h_{SD,RD,SR}}^{LS} = {{\hat{h}}}_{SD,RD,SR} - h_{SD,RD,SR}$, and the error variance of ${{\hat{h}}}_{SD,RD,SR}$ can be expressed as

9a $$\begin{aligned} \left| \varepsilon _{SD}\right| ^2&=\frac{\left( 1+d_{SD}^e\right) \sigma ^4}{P_sa_1x_1^2+P_sa_2x_2^2+2P_s\sqrt{a_1a_2}x_1x_2}, \end{aligned}$$

9b $$\begin{aligned} \left| \varepsilon _{SR}\right| ^2&=\frac{\sigma ^4\left( 1+d_{SR}^e\right) }{P_sa_1\alpha x_1^2\left[ p\right] +P_sa_2\alpha x_2^2\left[ p\right] +P_s\alpha \sqrt{a_1a_2}x_1\left[ p\right] x_2\left[ p\right] }, \end{aligned}$$

respectively. Therefore, the estimation error is ${{\widetilde{e}}}_{h_{SD,RD,SR}}^{LS}\sim \mathscr{C}\mathscr{N}\left( 0,\left| \varepsilon _{SD,RD,SR}\right| ^2\right)$.

10 $$\begin{aligned} \left| \varepsilon _{RD_m}\right| ^2=\frac{\Gamma _{D}+\Gamma _{E}+\Gamma _{F}}{\Gamma _{G}+\Gamma _{H}+\Gamma _{I}}. \end{aligned}$$

where $$\Gamma _{D}=\sigma ^4(1+d_{RD_m}^e){(1+d_{SR_m}^e)}^2P_s^2\left| h_{SR_m}\right| ^4$$, $$\Gamma _{E}=\sigma ^8(1+d_{RD_m}^e){(1+d_{SR_m}^e)}^4$$, $$\Gamma _{F}=2\sigma ^6(1+d_{RD_m}^e)P_s{(1+d_{SR_m}^e)}^3$$, $$\Gamma _{G}=\eta ^3{P_s}^4\left| h_{SR_m}\right| ^8\beta _{SR}^3(1-\alpha _{SR}-\beta _{SR})\left( x_1+x_2\right) ^2$$, $$\Gamma _{H}=\eta ^3{P_s}^3(\left| h_{SR_m}\right| ^6\beta _{SR}^3\sigma ^4$$, and $$\Gamma _{I}=\left| h_{SR_m}\right| ^7\beta _{SR}^3\sqrt{P_s(1-\alpha _{SR}-\beta _{SR})}\sigma ^2\left( x_1+x_2\right) )$$.

### Relay selection

To minimize the complexity of the system, select an optimal relay location from multiple relay nodes. Each relay can be in one of two states: cooperation or noncooperation. This implies that there are $2^N-1$ possibilities. Excluding a specific situation where all relay nodes are in a “uncooperative” state. This is because if all relay nodes do not cooperate, then in reality no relay node is working, which is often considered invalid or not considered in practical applications.

By employing the relay selection technique, the relay nodes with an equivalent SNR greater than the interruption probability threshold for the corresponding relay link are included in the candidate relay set initially. Then, the SNR is calculated for the relays in the set, and this information is fed back to the source node. The source node selects an appropriate relay as the data forwarding node based on the relay selection method and informs the relay selection result to each relay node.

In the following, we employ two relay selection schemes for contrast.

- Optimal relay selection (ORS) The ORS policy is based on the capacity expression achieved, and the criterion can be expressed as
  11 $$\begin{aligned} {\widetilde{k}}=arg\mathop {\max }_{{k\in \left\{ 1,\cdots , N\right\} }} {\left\{ \frac{\gamma _{SR_m}\gamma _{RD_m}}{\gamma _{SR_m}+\gamma _{RD_m}+1}\right\} }. \end{aligned}$$
- The max–min relay selection (MMRS) The conventional ORS policy does not take into account loop interference and selects *k*th relay. The criterion for this type of relay selection can be obtained as follows
  12 $$\begin{aligned} {\widetilde{k}}=arg\ \left( \text {max}\left\{ \text {min}\left\{ \gamma _{SR_m},\gamma _{RD_m}\right\} \right\} \right) . \end{aligned}$$
  The diversity multiplexing trade-off for this scheme is analyzed in^17^ based on the outage probability. In the above two methods, the destination node makes the selection and notifies the selected relay node, instead of computing and feeding back the power allocated to every relay node, which has less complexity and higher throughput than the relay selection method for power allocation.

### Outage performance analysis

In the case of the independent identically distributed (i.i.d.) SINRs, the best relay in the multi-relay network is selected, which can provide the largest end-to-end SINR for forwarding signals. Consequently, the outage probability of the relay selection scheme for *N* relay networks is

13 $$\begin{aligned} P_*=\text {P}_\text {r}\left\{ {\text {log}}_2\left( 1+\frac{\gamma _{SR_m}\gamma _{RD_m}}{\gamma _{SR_m}+\gamma _{RD_m}+1}\right) <R_0\right\} , \end{aligned}$$

where $*$ refers to different relay selection methods such as ORS and MMRS, and $\text {P}_\text {r}$ denotes the outage probability. In the following subsections, the statistical distributions may differ depending on the selection policy. Therefore, any remark concerning the distributions of these random variables (RVs) is strictly limited to the particular selection policy.

- Outage analysis of ORS By applying a straightforward order statistic result to Eq. (13), we can derive the following
  14 $$\begin{aligned} P_{\text {ORS}}=\text {P}_\text {r}\left\{ \frac{\gamma _{SR_m}\gamma _{RD_m}}{\gamma _{SR_m}+\gamma _{RD_m}+1}<x_{ref}\right\} , \end{aligned}$$
  where $x_{ref}=2^{R_0}-1$, the cumulative distribution function (CDF) of $\gamma _{RD_m}$ is $F_{\gamma _{RD_m}}\left( x\right) =1-e^{-\lambda _{RD_m}x}$ with $\lambda _{RD_m}=\frac{1}{\gamma _{RD_m}}$, $F_{\gamma _{SR_m}}\left( x\right) =1-e^{-\lambda _{SR_m}x}$ is the CDF of $\gamma _{SR_m}$ with $\lambda _{SR_m}=\frac{1}{\gamma _{SR_m}}$, $f_{\gamma _{RD}}\left( y\right)$ is the probability density function (PDF) of $\gamma _{RD}$, and the CDF of the RVs of $\gamma _i$ can be obtained as
  15 $$\begin{aligned} \begin{aligned} F_i\left( x\right)&=F_{\gamma _{RD}}\left( x\right) +\int _{x}^{\infty }F_{\gamma _{SR}}\left( \frac{\left( y+1\right) x}{y-x}\right) f_{\gamma _{RD}}\left( y\right) dy \\&=1-\lambda _{RD_m}a^{x}\sqrt{bx\left( x+1\right) }K_1(\sqrt{cx\left( x+1\right) }). \end{aligned} \end{aligned}$$
  where $a=e^{-\lambda _{RD_m}-\lambda _{SR_m}}$, $b=\frac{4\lambda _{SR_m}}{\lambda _{RD_m}}$, $c=4\lambda _{SR_m}\lambda _{RD_m}$, and K-function is a generalization of the hyper-factorial to complex numbers. The outage probability of ORS in the cooperative-NOMA system is
  16 $$\begin{aligned} P_{\text {ORS}}=\left[ F_i\left( x\right) \right] ^N. \end{aligned}$$
- Outage analysis of MMRS In the case of MMRS, $P_*$ can be written as
  17 $$\begin{aligned} P_{\text {MMRS}}=1-\int _{0}^{\infty }\left[ 1-F_{\gamma _{SR_m}}\left( \frac{xy+x^2+x}{y}\right) \right] f_{\gamma _{RD_m}}\left( y+x\right) dy, \end{aligned}$$
  For *N* relays that are independent of each other, according to the significance of $F_{\gamma _{SR_m}}$, we can deduce the CDF of $\gamma _{SR_m}$ as
  18 $$\begin{aligned} F_{\gamma _{SR_m}}(x)&=N\int _{0}^{x}\Pr {\left\{ k=i\mid \gamma _{{SR_m}_i}=y\right\} f_{\gamma _{{SR_m}_i}}\left( y\right) }dy, \end{aligned}$$
  In the MMRS method, the choice is based on the inferior of $\gamma _{SR_m}$ and $\gamma _{RD_m}$. Therefor $Pr\left\{ k=i\mid \gamma _{{SR_m}_i}=y\right\}$ can be further divided into two mutually exclusive cases $\gamma _{SR_m}>\gamma _{RD_m}$ and $\gamma _{SR_m}<\gamma _{RD_m}$. Finally, after a series of complex simplifications, $F_{\gamma _{SR_m}}(x)$ can be written as^18^
  19 $$\begin{aligned} \begin{aligned} F_{\gamma _{S R}}(x)&=N \frac{\left( 1-e^{-\lambda _{S R} x}\right) }{\lambda _{S R}+\lambda _{R D}} \sum _{n=0}^{N-1} \frac{(-1)^n\left( \begin{array}{c} N-1 \\ n \end{array}\right) }{\frac{n}{\lambda _{R D}}+\frac{1}{\lambda _{S R}+\lambda _{R D}}} \\&\quad -\frac{N \lambda _{S R}}{\lambda _{S R}+\lambda _{R D}} \sum _{n=0}^{N-1} \frac{(-1)^n\left( \begin{array}{c} N-1 \\ n \end{array}\right) \left( 1-e^{-(n+1)\left( \lambda _{S R}+\lambda _{R D}\right) x}\right) }{\left( \frac{n}{\lambda _{R D}}+\frac{1}{\lambda _{S R}+\lambda _{R D}}\right) (n+1)\left( \lambda _{S R}+\lambda _{R D}\right) } \\&\quad +N \lambda _{S R} \sum _{n=0}^{N-1} \frac{(-1)^n\left( \begin{array}{c} N-1 \\ n \end{array}\right) \left( 1-e^{-(n+1)\left( \lambda _{S R}+\lambda _{R D}\right) x}\right) }{(n+1)\left( \lambda _{S R}+\lambda _{R D}\right) } \end{aligned} \end{aligned}$$
  where $F_{\gamma _{RD_m}}(x)$ is obtained in the same way as $F_{\gamma _{SR_m}}(x)$, with CDF of $\gamma _{RD_m}$, we can obtain the PDF of $\gamma _{RD_m}$. Hence, substitute $F_{\gamma _{SR_m}}(x)$ and $f_{\gamma _{RD_m}}$ into Eq. (17) to evaluate the exact outage probability using numerical integration. Therefore, the interruption probability of the entire system can be expressed as
  20 $$\begin{aligned} P_I=1-\left( 1-P_*\right) \left( 1-P_r\left\{ R_{SD_{m}}<R_{th1}\right\} \right) . \end{aligned}$$
  The Eq. (20) represents the overall system interruption probability, where $P_*$ denotes the probability of interruption in the relay link, and $P_r\left\{ R_{SD_{m}}<R_{th1}\right\}$ signifies the probability of interruption in the direct link when the data rate $R_{SD_{m}}$ falls below the threshold $R_{th1}$. This expression effectively captures the likelihood that the system will experience an interruption due to either a failure in the relay link or insufficient performance in the direct link, considering these two events independently. In our analysis, the impact of channel estimation error was implicitly included through the variance of the estimation error in the signal-to-noise ratio (SNR) expressions used to calculate the outage probability. Specifically, the SNR at the relay and the destination already accounts for the degradation due to the estimation error introduced by the Least Squares (LS) method.

## Data Availability

URL The authors confirm that the data supporting the findings of this study are available within the URL https://github.com/zzzzzzzzzzzzzzy/Analysis-of-the-OutagePerformance-of-Energy-Harvesting-Cooperative-NOMA-System-with-Relay-Selection.
